# The Infant and Toddler Curiosity Questionnaire: A Validated Caregiver‐Report Measure of Curiosity in Children From 5 to 24 Months

**DOI:** 10.1111/infa.70001

**Published:** 2025-01-23

**Authors:** Elena C. Altmann, Marina Bazhydai, Didar Karadağ, Gert Westermann

**Affiliations:** ^1^ Department of Psychology Lancaster University Lancaster UK

**Keywords:** early development, exploration, individual differences, infant curiosity, psychometrics, trait curiosity

## Abstract

Humans are curious. Especially children are known for their drive to explore and learn, which is crucial for developing in and navigating through our complex world. Naturally, some children may be more curious than others, leading to differences in how they structure their own learning experiences, subsequently impacting their developmental trajectories. However, there is a gap in the research field for a reliable measure of such differences early in development. Across three studies, we present the development and assessment of the Infant and Toddler Curiosity Questionnaire (ITCQ), the first caregiver report measure to fill this gap. Items cover observable exploration behaviors in 5‐ to 24‐month‐olds to capture general tendencies of their desire to actively explore their immediate surroundings and are evaluated on a 7‐point Likert‐scale. Exploratory factor analyses and structural equation modeling on a sample of *N* = 370 UK caregivers led to the final selection of 23 items and provided evidence that the scale allows the reliable computation of an overall curiosity score, with three emergent subscales (*Sensory, Investigative*, and *Interactive*) explaining additional variance in the data. Furthermore, the scale had good test‐retest reliability after 7–14 days (*N* = 67) and related to the child's temperament (*N* = 75; positively with surgency and effortful control, negatively with negative affect) offering evidence of its validity as a trait measure. Together, these results support the scale's reliability and validity, showcasing the ITCQ as a powerful tool for developmental research.

## 1 Introduction

While the study of curiosity and its effects on learning has a long history in adults (Berlyne 1960; Gruber, Gelman, and Ranganath 2014; Kang et al. 2009; Rossing and Long 1981), only in recent years has infant curiosity become a focus of research and has provided insights into how infants actively engage in their own learning. Studies have shown, for example, that infants prefer to engage with information of intermediate complexity (Kidd, Piantadosi, and Aslin 2012, 2014) and that they alternate between visual exploration and exploitation driven by their active learning experience (Altmann, Bazhydai, and Westermann 2025) in the pursuit of maximizing their learning progress (Poli et al. 2020; Twomey and Westermann 2018), that they actively request information from adults through social orienting (Bazhydai, Westermann, and Parise 2020) and pointing (Liszkowski, Carpenter, and Tomasello 2007), with learning benefits shown for actively requested information (Begus, Gliga, and Southgate 2014) but also from being in a state of curiosity more generally (Chen, Twomey, and Westermann 2022; Stahl and Feigenson 2015). What is common to all of these studies is that they assume infants to be curious learners by definition and that they investigate the implications of such inherent trait curiosity on in‐the‐moment behaviors. Only a few studies, however, have considered how differences in infants' individual interests (Ackermann, Hepach, and Mani 2020) and sensory seeking (Piccardi, Johnson, and Gliga 2020) affect their preference to engage with specific information. Overall, there has been no systematic investigation on individual differences in trait curiosity on infants' exploratory behavior, learning, and later outcomes. To enable such research, it is necessary to have validated measures of infant curiosity.

This is different from research in adults where multiple scales exist to assess variation in trait curiosity (for reviews, see Grossnickle 2016; Jirout and Klahr 2012; Wagstaff et al. 2021), measuring general accounts of curiosity (e.g., Day 1971; Kashdan et al. 2020; Litman and Jimerson 2004; Litman and Spielberger 2003; Naylor 1981; Spielberger 1979) but also more specific domains (e.g., social curiosity, Renner 2006; work‐related curiosity, Mussel et al. 2012) in the form of self‐report questionnaires. These measures typically ask responders how commonly or intensely they experience a desire for knowledge and learning, thereby requiring meta‐cognitive awareness (e.g., Goupil and Proust 2023; Loewenstein 1994). However, some questionnaires also conceptualized curiosity as the intrinsic motivation behind exploration in the pursuit of knowledge, leading to items focusing on more observable behaviors (e.g., Kashdan et al. 2009). Research using such trait measures has shown positive associations between curiosity and job performance as well as academic achievement (e.g., Grossnickle 2016; Hardy, Ness, and Mecca 2017; Kashdan and Yuen 2007; Mussel 2013; Reio and Wiswell 2000; Reio and Callahan 2004), highlighting its impact on life outcomes.

To investigate trait curiosity in children, some self‐report measures designed for school‐aged cohorts do exist (Byman 2005; Maw and Maw 1968; Olson 1986; Penney and McCann 1964) but their validity may be limited by the children's lack of motivation to self‐reflect and reliably answer numerous repetitive items (Jirout and Klahr 2012). An alternative to self‐reports, especially relevant for younger children, are *other*‐reports (primarily caregivers and teachers, e.g., Harty and Beall 1984; Lee et al. 2023; Maw and Maw 1970; Piotrowski, Litman, and Valkenburg 2014). Other‐reports enable measurement without age‐restriction but do require another person to assess a latent (not directly observable) construct in the child, bringing its own challenges. Yet, extensive research from infancy onward has shown that such measures can generate reliable, valid, and longitudinally informative data by, for instance, having items address observable behaviors in which the latent construct manifests itself. Prominent examples include the Ages and Stages questionnaire (e.g., Klamer et al. 2005; Lépine et al. 2022; Richter and Janson 2007; Salomonsson and Sleed 2010), the MacArthur‐Bates Communicative Development Inventory (CDI; Bornstein and Putnick 2012; Can et al. 2013; Feldman et al. 2005; Fenson 2002; Fenson et al. 1994, 2006; Marchman and Fernald 2008), and temperament scales from infancy to early childhood (e.g., Putnam, Rothbart, and Gartstein 2008; Rothbart 1986, 2011; Slagt et al. 2016; Wright and Jackson 2022). However, there is a clear gap in the scientific literature regarding infant trait curiosity. Even though some emerging work has aimed to assess differences in early curiosity through caregiver reports (Lee et al. 2023; Piotrowski, Litman, and Valkenburg 2014) the target group of infants and toddlers has thus far been neglected.

As mentioned, for other‐reports it is important to create items based on observable behavior in which the construct manifests. Regarding curiosity, this manifestation is commonly assumed to be active exploration and interaction with the environment (for review see Bazhydai, Twomey, and Westermann 2020; Jirout, Evans, and Son 2024). Previous research across the first 2 years of life has found individual differences in exploration throughout various experimental paradigms, such as visual exploration (e.g., Colombo et al. 1991; Franchak et al. 2016; Piccardi, Johnson, and Gliga 2020; Wass and Smith 2014), manual exploration (e.g., Fortner‐wood and Henderson 1997; Mandler, Fivush, and Reznick 1987; Muentener, Herrig, and Schulz 2018) and free play exploration (e.g., Bornstein, Hahn, and Suwalsky 2013; Slone, Smith, and Yu 2019; Smith and Yu 2013), letting us plausibly assume that infants already differ in their trait curiosity. Some of these studies also found that exploration differences were predictive of variability in learning, later vocabulary, cognitive development, and academic achievement (e.g., Berg and Sternberg 1985; Bornstein, Hahn, and Suwalsky 2013; Muentener, Herrig, and Schulz 2018; Smith and Yu 2013) highlighting its role in and importance across development. In fact, one study (Shah et al. 2018) used a subset of five caregiver‐report items as an ad hoc measure of curiosity (e.g., “Likes to try new things.”, “Shows eagerness to learn new things.”) and was able to find a positive relation with academic achievement in kindergarten. It is to be noted, however, that the other three included items rather capture skills *related* to curiosity, such as metacognitive communication (“Appropriately uses a variety of words to describe feelings.”), creativity (“Shows imagination in work and play.”) and temperament (“Easily adjusts to a new situation.”), but not curiosity directly. Thus, it is important to stress that measures used in psychological research need to be reliably constructed and validated with regards to the construct they aim to capture (e.g., Flake and Fried 2020). Nevertheless, this finding does hint at the impact a systematically developed and validated caregiver report measure of curiosity could have. A gap in the literature remains regarding a measure which can capture individual differences in curiosity through active exploration tendencies in pre‐verbal infants.

### 1.1 The Current Paper

We present the Infant and Toddler Curiosity Questionnaire (ITCQ) applicable for 5–24‐month‐old infants and toddlers as a new caregiver‐report measure for capturing individual differences in trait curiosity. Considering the lack of consensus for a functional definition of curiosity especially early in development (Jirout and Klahr 2012; Kidd and Hayden 2015), we decided to base our approach on a folk psychology definition of infant curiosity, that is, *a keen desire or tendency to actively explore one's immediate surroundings*. Here we report three studies on the ITCQ's development and assessment, evidencing its reliability and validity in line with rigorous practices (e.g., Downing 2003; Flake and Fried 2020; Messick 1995). Study 1 describes the questionnaire development, including its content and structural validity (sample size and general analytical approaches were pre‐registered at https://aspredicted.org/19J_291). Study 2 supports the ITCQ's test‐retest reliability after 7–14 days, and study 3 explores the measure's criterion validity via its relation to the well‐established trait measure of temperament. Studies were given ethical approval by the University Faculty's research ethics committee, and data as well as analysis scripts are available on the Open Science Framework (OSF).

## 2 Study 1: Questionnaire Development & Structural Validity

In this first study we describe the principles underlying the ITCQ's creation, a reduction of items to generate coherent and reliable responses, as well as offering evidence for its content and structural validity (Downing 2003; Messick 1995). Content validity concerns whether the items are representative and well formulated, and whether ambiguities were resolved, which was largely the focus of the item development and an initial pilot study. Structural validity, on the other hand, concerns the scale's dimensionality and internal consistency, which was the focus of the main analysis of this study. While we created the items with the purpose of measuring a single factor of general curiosity, it is recommended for new scales to explore and consider the best fitting emerging factor structure to explain additional variance in the data (e.g., McCoach, Gable, and Madura 2013). Furthermore, we aimed to provide evidence supporting our intention of one general factor (Artino et al. 2014) to justify the computation of an overall mean score (Dunn and McCray 2020).

### 2.1 Questionnaire Development

We developed an initial set of 34 statements capturing a wide range of behaviors infants can produce to interact with their physical and social environment as the manifestation of their curiosity. For this, we reviewed scientific and general sources (e.g., parent forums) regarding exploration behaviors children typically express throughout their first 2 years of life (looking, grabbing, mouthing, pointing, etc. typically directed at objects; Adolph and Hoch 2019; Lockman 2000) as well as everyday situations and locations in which they could be observed (e.g., at home or in new environments). Furthermore, we avoided focusing on curiosity about new people as this special domain of curiosity may be too heavily influenced by differences in temperament (e.g., shyness). Based on these sources, various items were proposed, and those deemed to capture the construct best by the research team were eventually selected. This process resulted in a list of 34 items which allowed for a diverse selection of behaviors while only requiring a manageable amount of time and effort from responders. These items covered behaviors such as interacting with objects, enjoyment of new discoveries, and interactions to gain information (for a full list of final items, see Table 1).

**TABLE 1:** Final questionnaire items (plus three excluded items), their descriptives (mean, standard deviation, proportion NA, and item difficulty), and exploratory factor loadings.

Item		*M (SD, Median)*	NA	Difficulty	General factor	3‐Factor model		
						1	2	3
S1	When my child encounters an object, they typically seem interested in its properties (e.g., how it feels, tastes, or sounds like, etc.)	6.2 *(0.81, 6)*	0.00	0.88	0.34	**0.61**		
S2	My child actively inspects a variety of objects, whether it be toys or ordinary household items	6.6 *(0.63, 7)*	0.00	0.94	0.40	0.50		
S3	My child usually inspects objects from all angles and sides	5.6 *(1.15, 6)*	0.01	0.80	0.44	0.51		
S4	My child pokes at and probes objects to see how they feel	6.0 *(1.01, 6)*	0.02	0.86	0.43	0.51		
S5	My child is interested in a wide variety of objects	6.5 *(0.70, 6)*	0.00	0.93	0.41	0.52		
S6	My child actively seeks out and enjoys new experiences	5.8 *(1.05, 6)*	0.05	0.83	0.53	0.45		
S7	My child shows visible enjoyment (e.g., smiling, gurgling, babbling) when discovering something new	6.3 *(0.83, 6)*	0.00	0.90	0.40	0.44		
S8	My child enthusiastically explores new environments (e.g., a new house, the beach, etc.)	5.9 *(1.10, 6)*	0.08	0.85	0.47	0.43		
S9	My child likes to look around, scanning the environment for something new	6.1 *(0.94, 6)*	0.00	0.87	0.39	0.39		
S10	My child is interested in what other people next to them are doing. For example, when someone prepares food, my child closely observes their every move	6.0 *(1.00, 6)*	0.02	0.86	0.42	0.37		
SR	My child does ***not*** typically engage with (look at, point at, reach for, inspect) a lot of things in their environment	6.4 *(0.88, 7)*	0.01	0.91	0.43	0.36		
Iv1	When my child looks into a container (e.g., a bag, kitchen drawer, etc.), they take out and inspect each of its contents	6.1 *(1.10, 6)*	0.06	0.87	0.57		**0.70**	
Iv2	When something is hidden from my child (e.g., in closed boxes, rooms, cupboards etc.), they will actively try to uncover it	6.0 *(1.21, 6)*	0.09	0.86	**0.64**		0.66	
Iv3	When I open my bag in front of my child, they will come and peek into it	6.1 *(1.15, 6)*	0.11	0.87	0.54		0.64	
Iv4	My child often bangs objects to see what noise they make	6.3 *(1.10, 7)*	0.02	0.89	0.32		0.40	
Iv5	If a toy has multiple functions, my child will typically discover and play with more than one of them	5.6 *(1.28, 6)*	0.05	0.80	0.62		0.33	
Ia1	My child often leads me to/brings me things that they want to know more about	5.2 *(1.56, 6)*	0.21	0.75	0.55			**0.77**
Ia2	When reading a picture book together, my child directs me (e.g., by pointing) toward what they want to know more about	4.7 *(1.87, 5)*	0.19	0.68	0.54			0.71
Ia3	When we are in a new environment (e.g., the zoo, a shop, etc.), my child keeps pointing at all the things they find interesting	5.1 *(1.87, 6)*	0.18	0.73	0.53			0.66
Ia4	When faced with a problem, my child will seek the help of others in order to solve it	4.9 *(1.41, 5)*	0.15	0.70	0.46			0.61
Ia5	When faced with a problem (e.g., fitting a block into its respectively shaped hole), my child typically keeps trying to figure it out until they have solved it	4.6 *(1.60, 5)*	0.16	0.66	0.53			0.38
Ia6	When someone shows my child how something works, they watch with continuous interest	5.7 *(1.06, 6)*	0.04	0.81	0.54			0.36
*Ia7*	*When my child is confused by something, they look at me/another person for additional information*	*5.7 (0.98, 6)*	*0.06*	*0.82*	*0.55*			
*Ex1*	*My child starts playing on their own, rather than waiting to be given something to play with*	*6.1 (1.01, 6)*	*0.02*	*0.87*	*0.32*			
*Ex2*	*When my child plays with an assembly toy (e.g., building blocks, puzzle, a toy with detachable parts), they like to take it apart for further examination*	*5.8 (1.25, 6)*	*0.13*	*0.83*	*0.51*			
*Ex3*	*When playing hide and seek, my child enjoys searching for the object or person that disappeared*	*6.2 (0.87, 6)*	*0.16*	*0.88*	*0.42*			

*Note:* Items are ordered by subfactors and loadings on those subfactors (S = Sensory, Iv = Investigative, Ia = Interactive, Ex = Excluded). Item difficulty captures the likelihood of receiving the maximum score so that higher values indicate lower difficulty (ideally between 0.5 and 0.8 which is only given for the third subfactor). “NA” indicates the sample proportion of NA responses. Factor loadings smaller than 0.32 are not reported, the strongest loading per factor is marked in bold. Italicized items are those that loaded onto the general factor but did not load sufficiently onto any sub‐factor.

Due to the necessarily developmental perspective, some behaviors may not yet be observable in younger infants such as interacting socially (e.g., “When reading a picture book together, my child directs me [e.g., by pointing] toward what they want to know more about.”) whereas other items were expected to be equally applicable across ages (e.g., “When my child encounters an object, they typically seem interested in its properties [e.g., how it feels, tastes or sounds like, etc.]”). To constrain the variance in applicability of items, we decided to focus on an age range from five to 24 months. The minimum of 5 months was chosen based on a notable expansion in behaviors infants can produce from this age, whereas 24 months was chosen as the upper limit because from around the second birthday onwards verbal expressions of curiosity, such as question asking, become more prevalent.

Three of the items were reverse coded and described non‐curious behavior (e.g., “My child does not typically engage with [look at, point at, reach for, inspect] a lot of things in their environment.”). While it is recommended to include such items to enhance data quality by making the reader slow down (Boley, Jordan, and Woosnam 2021), they can also reduce the scale's overall reliability due to inattentive responses and lack of clarity (Rossiter 2002; Salazar 2015; Weijters and Baumgartner 2012), often leading to their exclusion during the structural validity investigations. With this in mind, we included two additional items which were the mirrored versions of other positive items, solely meant to increase the responders' attention but not to be analyzed as part of the final dataset (see Supporting Information S1: Section S1.1). Even though they are not considered part of the final item list, they can be optionally included (e.g., for larger online studies with potentially lower response quality).

We chose a 7‐point Likert‐scale from 1 (“strongly disagree”) to 7 (“strongly agree”) as the response scale, with an option of “not applicable (NA)” if respondents could not think of any recent situation allowing them to rate a specific item or because their child had not yet been able to show the behavior. Items were created in English initially targeting British caregivers and were repeatedly reviewed and improved based on the topical expertise of the authors, as well as through discussions with parents and native speakers to ensure their content validity. For further considerations regarding the response scale and online presentation, see Supporting Information S1: Section S2.

### 2.2 Piloting

A pilot sample (*N* = 22, age in months: *M* = 11.5, SD = 1.6, 41% female; £5 travel reimbursement given), collected from primary caregivers (all mothers) participating in an in‐person study with typically developing 10‐ to 12‐month‐olds in the north‐west of England, provided the first support for the questionnaire's construction. The exclusion of two items was suggested to improve the scale's homogeneity, without which the measure had very good coefficients commonly used to indicate internal consistency (Coefficient alpha = 0.87, lambda‐6 = 0.93). This offered first evidence that the scale was constructed sensibly enough to continue wider data collection (Nunnally and Bernstein 1994).

At the end of the survey, caregivers were also invited to provide qualitative responses of additional behaviors capturing curiosity. These responses were found to reflect very similar behaviors and situations already covered in the questionnaire items (e.g., being interested in how things feel, trying to see what objects are on the table, etc.). They also supported our conceptualization of curiosity being in line with how parents intuitively understand the construct; thus together, these findings evidenced the scale's content validity. In‐person comments received from caregivers after completing the questionnaire offered additional insights as they mentioned that the questionnaire was clearly formulated and easy to complete. Some parents mentioned that the items let them easily differentiate between behavioral tendencies of their youngest child and their older siblings which they found fascinating.

Due to these overall promising preliminary results, we continued with wider data collection across the full age‐range making no changes to the scale (i.e., also not removing the two ill‐fitting items at this point in case their effect was due to the constrained sample). Based on this decision, the pilot data was deemed suitable to be included in the following main analyses.

### 2.3 Methods

#### 2.3.1 Participants

**FIGURE 1:**
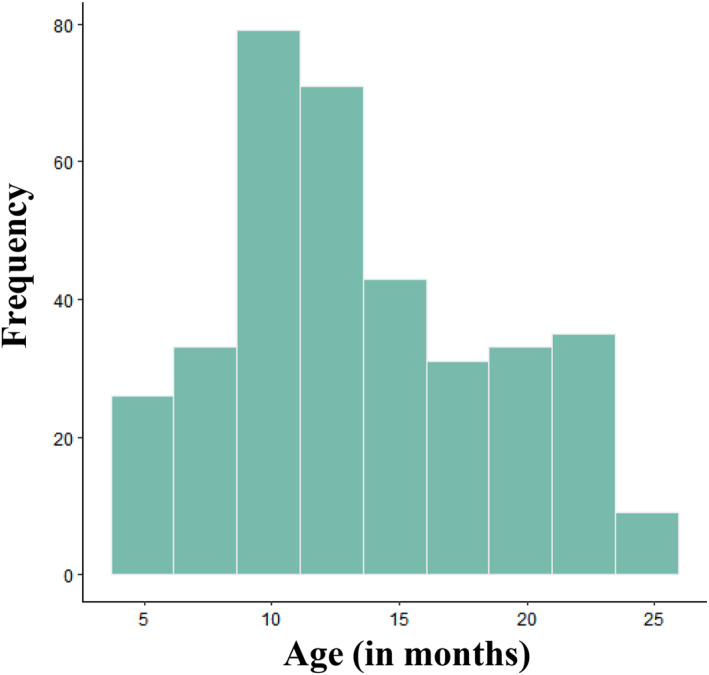
Age distribution of the full sample.

A minimum sample size of *N* = 360 was preregistered following a rule of thumb with 10 participants per item (e.g., Nunnally 1978). A total of *N* = 370 responses were included in the final analyses (age range in months: 4.5–24.4, *M* = 13.5, SD = 5.2, see Figure 1; 51% female). Of these, *n* = 243 were recruited via social media, *n* = 72 attended in‐lab visits, and *n* = 54 were contacted from the Babylab's database (which includes contact details for families willing to take part in infancy studies in the north‐west of England) to directly complete the questionnaire alongside a temperament questionnaire (see study 3). Fifty‐five additional responses were excluded due to being outside the preregistered age‐range (*n* = 16), not being from the UK (*n* = 5), prematurity (*n* = 17), developmental concerns (*n* = 14), or poor data quality (*n* = 2, where all responses were either NA or the exact same response including reverse coded items). From the final sample, 97% indicated their child to be monolingual English, 82% of caregivers (predominantly mothers as per social media engagement, database recruitment and direct communication) indicated to have achieved a degree in higher education (e.g., bachelor's degree and above), 50% of children were said to be the first born, 40% second born, and 10% were reported to have at least 2 older siblings (including stepsiblings). Participants recruited online via social media were invited to complete the survey without reimbursement whereas the sub‐samples directly recruited from the Babylab's database received reimbursement as per university guidelines: in‐lab visiting participants received £5 for their travel, and the sample which completed the longer version including the temperament scale received a £5 online gift voucher of their choice (via express.giftpay.com). The reported studies (1–3) were conducted according to guidelines laid down in the Declaration of Helsinki, with written informed consent obtained from a parent or guardian for each child before any assessment or data collection. All procedures received ethical approval under FST21068 at Lancaster University.

#### 2.3.2 Materials

Aside from the piloted 34 items, the questionnaire also included two items directly addressing the construct: an item asking about the child's curiosity directly (“I would describe my child as curious”) and one item about the child's curiosity in comparison to their peers (on an ad hoc five‐point scale from 1 [“a lot less curious”] to 5 [“a lot more curious”]). Additionally, respondents were asked to provide demographic information (prematurity, developmental concerns, country, languages spoken, birth order, and socio‐economic status [SES] via their educational level; Singh et al. 2023), and could optionally contribute qualitative responses regarding additional behaviors in which they saw their child's curiosity manifested (exact items in Supporting Information S1: Section S1.3).

#### 2.3.3 Procedure

Primary caregivers across the UK were invited to complete this online questionnaire on Qualtrics (Qualtrics, Provo, UT). Participating caregivers were provided with an information sheet before giving informed consent and received instructions to think about their child's typical behavior to evaluate each statement. They indicated their child's age and sex after which the items were presented in a randomised order with four to five items per page. These were followed by the two items assessing direct curiosity, optional qualitative responses, and demographic questions. Lastly, caregivers were given the opportunity to sign up to receive an automatic email a week later for a re‐test. It took most respondents under 15 min (*M* = 11.43, SD = 5.45) to complete the survey.

#### 2.3.4 Analyses

All analyses were conducted in R (Version 4.1.2).

##### 2.3.4.1 Item Reduction and Emerging Sub‐Factors

The scale was designed to measure infants' and toddlers' trait curiosity as one general construct represented in the tendencies with which the infant explores their surroundings. Thus, the aim of the exploratory factor analysis was to reduce the item list to coherently capture this construct, as well as to better understand the additional variance in the data. This analysis required initial steps (following Pett, Lackey, and Sullivan 2003) of identifying possibly ill‐fitting items and assessing the scale's sampling adequacy and factorability. We then fitted a unidimensional structure eliminating items which did not sufficiently load onto the general curiosity factor (< 0.32). Subsequently, we explored how many sub‐factors the items grouped into to further investigate the scale's dimensionality (Dunn and McCray 2020), where the number of sufficient factors was indicated using a scree plot and a parallel analysis. For the two latter steps, we conducted Exploratory Factor Analyses (function “fa” from the *psych* package) using default minimum residual factoring (*minres*) and oblique rotation allowing for sub‐factors to correlate. The *minres* factoring method is recommended for questionnaire data (Fabrigar et al. 1999) since it gives robust estimates even for skewed items, whereas correlation among emerging sub‐factors was justified by our theoretical assumption of one underlying construct.

##### 2.3.4.2 Structural Validity

Having multiple items can lead to an emergent sub‐factor structure if item topics and wordings result in correlated response behavior. The current scale, however, was constructed to measure a general factor of trait curiosity due to lacking a strong theoretical basis for assuming multi‐dimensionality of curiosity in early childhood. Here, we explored the scale's dimensionality to support the computation of an overall curiosity mean score (Dunn and McCray 2020) by fitting the previously identified sub‐factor structure using Structural Equation Modeling (SEM; function “sem” from the *lavaan* package; Rosseel 2012). This allowed us to compare a unidimensional model to a correlational sub‐factors model. Note that we initially also fitted a bi‐factor model which, however, was considered less interpretable and was consequently removed from this report. Due to the expected occurrence of missing values (N/A responses for not yet or not recently observed behaviors), models were estimated using the robust full information maximum likelihood (estimator = “MLR” in lavaan). Model comparison included standard indices such as chi‐square, comparative fit index (CFI), Root Mean Square Error of Approximation (RMSEA), and Bayesian Information Criterion (BIC).

##### 2.3.4.3 Internal Consistency

On the final set of items, commonly reported measures of internal consistency were computed, namely Coefficient alpha (also referred to as Cronbach's alpha, McNeish 2018), Revelle's Omega total, and Guttman's lambda‐6 (using “omega” from the *psych* package), as well as Revelle's coefficient Beta. Revelle's Beta estimates how much variation in the data can be attributed to some general underlying factor (Cooksey and Soutar 2006), so that a general factor can be argued at beta values above 0.50, whereas values above 0.70 are recommended (John and Roedder 1981; Revelle 1979; Rossiter 2002). Regarding the other three measures, values above 0.80 are considered good (but are said to be acceptable above 0.70 when not meant for diagnostic decisions; Pett, Lackey, and Sullivan 2003) and the average Inter‐item correlation is ideally between 0.20 and 0.40 (Piedmont 2014). Additionally, we mentioned item‐response‐theory in the pre‐registration, but this was omitted from this report due to limited informativeness above and beyond the here reported results.

##### 2.3.4.4 Exploration of Demographic Differences

We explored whether mean scores systematically differed across sex, age and SES (as approximated via the mother's education), to inform future applications of the scale, using multiple linear regression. Mean scores were computed on the final set of items, with unobserved items (N/A responses) not affecting individual scores.

First, however, structural equation models were tested in three steps regarding measurement invariance across sexes: the first step tests whether factor loadings are the same between groups (metric invariance), the second adds constraints on the intercepts (scalar invariance) and the third on residual variances (strict invariance). Invariance is achieved at each step if adding the respective constraints does not significantly worsen model fit. If it does, however, specific parameters need to be inspected and relaxed to achieve partial invariance, and their impact on observed scores considered and discussed (Vandenberg and Lance 2000).

Lastly, we computed correlations between the overall mean scores and the two additional curiosity items as initial indications of construct validity, expecting positive relationships.

### 2.4 Results

#### 2.4.1 Item Reduction and Emerging Sub‐Factors

**FIGURE 2:**
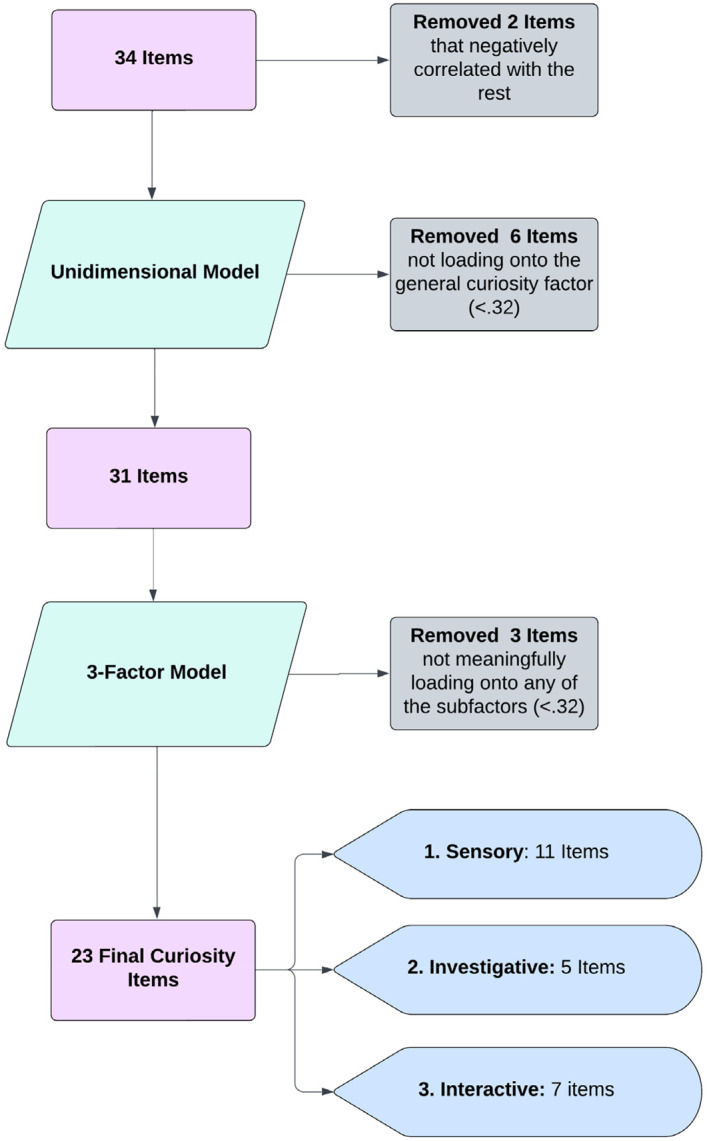
Schematic overview of the item reduction process.

A schematic overview of item reduction process is presented in Figure 2. We first reverse coded the three negatively formulated items and computed a correlation matrix (using Spearman's rho) to investigate ill‐fitting ones (e.g., Pett, Lackey, and Sullivan 2003). This led to the exclusion of two items which correlated negatively with many of the rest (new foods & mouthing; Supporting Information S1: Section S1.2). Then we ensured that the data was adequately sampled and factorable as indicated by a significant Bartlett's test of sphericity (*X* ^2^(496) = 3474.50, *p* < 0.001), a non‐zero, positive matrix's determinant (0.00006), and the Kaiser‐Meyer‐Olkin statistic above 0.70 for both, the overall sample (KMO = 0.89) as well as each individual item (lowest measure of sampling adequacy [MSA] = 0.82).

We then fitted a one‐factor model on the remaining 32 items to further reduce the item list to only those loading onto a general factor. In this way, six additional items (Supporting Information S1: Section S1.2) with loadings below the recommended 0.32 (Tabachnick and Fidell 2007) were eliminated. The subsequent exploration of emerging factors only included the 26 remaining items that loaded strongly and positively onto this general factor. Drawing a line through the lower values in the scree plot (Cattell 1966; Pett, Lackey, and Sullivan 2003; Figure S1) suggested 3 or 4 factors. Similarly, the parallel analysis indicated four factors, whereas the “elbow” of both graphs already occurred at factor 3. Accordingly, we fitted a 3‐factor and a 4‐factor model.

The 4‐factor model had overall better fit values (TLI = 0.89, RMSEA = 0.048, BIC = −921.22) than the 3‐factor model (TLI = 0.87, RMSEA = 0.052, BIC = −978.39). However, upon inspection of the emerging sub‐factors, both models were highly similar except for an additional two‐item factor in the 4‐factor model. As it has been argued that a meaningful factor should consist of at least three items (Hair, Black, and Babin 2010), we decided to continue with the 3‐factor model. Table 1 shows the items' descriptives and subfactor‐loadings in comparison to their loadings on the general factor. Overall, items were positively rated and only items on the third subfactor were within the ideal range of item difficulty. However, these items were also accumulating most NA responses indicating behaviors many infants had not recently or not yet expressed.

The first sub‐factor (11 items) could be labeled as *Sensory* as it includes items regarding more general manual and visual exploratory behaviors. The second subfactor (5 items) could be labeled as *Investigative* including items indicating a tendency to autonomously manipulate objects in their environment to gain information. The third subfactor (6 items) could be labeled as *Interactive* capturing to what degree the child uses and interacts with social partners to receive additional information. These labels aim to best describe each subfactor's contents especially considering the strongest loading items; but note item Ia5 as an exception.

Four items did not sufficiently load onto any of these subfactors. On inspection, three of these (Ex1, Ex2, Ex3) concerned play behavior more so than exploration which may explain their distinctness. However, item Ia7 had one of the strongest loadings toward the general factor and also loaded onto the Interactive subfactor in the 4‐factor model (at 0.41). As exploratory factor analysis is not meant to be a purely data‐driven process, we decided to keep this item given its strong contribution to the general factor and its contextual fit with the Interactive subfactor (now 7 items) while excluding the other three. Thus, all subsequent analyses were conducted on the final set of 23 items.

#### 2.4.2 Structural Validity

We fitted a unidimensional and a correlational structural equation model to investigate the scale's dimensionality and better understand how the items are structured. The model of three correlating subfactors had a significantly better model fit than the unidimensional model (∆*F*(3, 230) = 125.14, *p* < 0.001) yet was not meeting the general criteria of acceptable fits. Thus, we considered computationally suggested modifications with the top three being correlated residuals between items S6 and S8, S10 and Ia6, as well as Ia2 and Ia3. With these modifications, the correlational model showed acceptable fit measures (Table 2). The chi‐square test was significant in both models but this is to be expected at larger sample sizes (Schermelleh‐Engel, Moosbrugger, and Müller 2003; Vandenberg 2006). Expectedly, the subfactors were strongly correlated (Sensory ∼ Investigative: *r* = 0.64, Sensory ∼ Interactive: *r* = 0.51, Investigative ∼ Interactive: *r* = 0.75).

**TABLE 2:** Indices for the specified models using structural equation modeling (SEM).

Model	Chi‐square (df, *N*)	CFI^a^	RMSEA^b^	AIC^c^	BIC^d^	Adj. BIC^e^
Unidimensional	*χ* ^2^(230,370) = 703.98***	0.71	0.075 [0.069;.080]	22,468.58	22,738.61	22,519.69
Correlational	*χ* ^2^(224,370) = 388.63***	0.90	0.045 [0.038;.051]	22,098.38	22,391.89	22,153.95

*Note:* The correlational model includes the three modifications reported in structural validity section.

^a^ Comparative Fit Index (preferably ≥ 0.90).

^b^ Root Mean Square Error of Approximation (preferably ≤ 0.060) and [95% Confidence Intervals].

^c^ Akaike Information Criterion.

^d^ Bayesian Information Criterion.

^e^ Sample‐size adjusted BIC (all: smaller better).

***p < 0.001.

#### 2.4.3 Internal Consistency

We computed common measures of internal consistency for both the complete scale and the emergent subfactors (Table 3). The overall scale was found to have high internal consistency, where a Revelle's beta of > 0.70 additionally supports our assumption of a general underlying factor. Furthermore, the separate subfactors also had good (> 0.70) indices supporting these data‐driven options to explore additional variance in the sample. Consequently, we will refer to them as subscales.

**TABLE 3:** Measures of internal consistency for the full scale and the emergent subscales.

Scale	Number of items	Coefficient *α*	Lambda‐6	Revelle's omega total	Revelle's *β*
General	23	0.87	0.90	0.89	0.71
Sensory	11	0.78	0.78		
Investigative	5	0.74	0.71		
Social/interactive	7	0.81	0.80		

#### 2.4.4 Exploration of Demographic Differences

**FIGURE 3:**
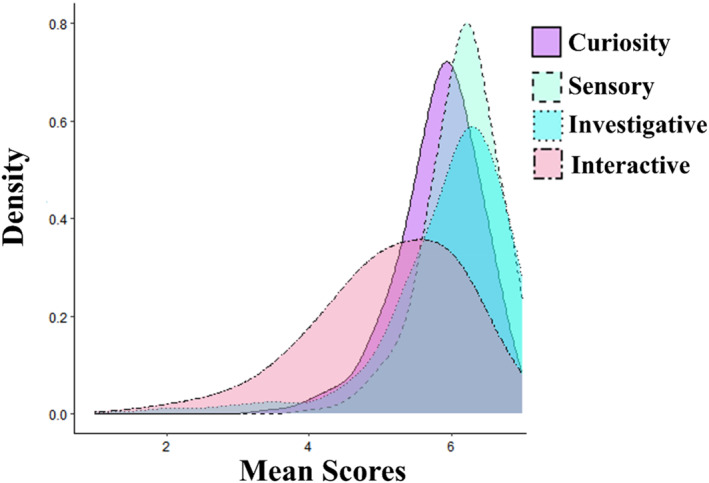
Mean score density plots for overall curiosity as well as the subscale scores.

The mean curiosity scores were distributed around an average of *M* = 5.83 (SD = 0.59), evidencing that the scale captures variance in reported exploration tendencies (Figure 3). To inspect demographic differences in mean scores, we first tested measurement invariance (metric, scalar, strict) across sex, that is, whether factor loadings, intercepts, and residual variances of each item in the correlational SEM model are the same for male and female infants. One factor loading (Sensory = ∼S2) was relaxed to achieve partial metric invariance, beyond which also scalar and strict invariance were achieved (all model comparisons *p* > 0.05, indicating that additional constraints did not worsen the model fit). The differing item S2 concerns inspection of various types of objects as part of the Sensory subscale. However, we see no concern for the computation and comparison of mean scores across sexes because of the following three reasons: (1) This item has limited impact as it is 1 of 11 items in that subscale, (2) it did not generate significantly different scores between sexes (*W* = 16,812, *p* = 0.812), and (3) because scalar invariance was achieved. Overall, this test supports the ITCQ's applicability across sexes.

Multiple linear regressions showed that age (*b* = 0.04, *p* < 0.001) but neither sex (*b*_*m*_ = 0.06, *p* = 0.305) nor SES (*b* = −0.02, *p* = 0.453) predicted curiosity scores (*F*(3, 324) = 18.05, *p* < 0.001). As we did not expect age to be a significant predictor, we exploratorily investigated these relations for each of the subscales and found the same pattern, while revealing that the age effect was mainly driven by the Interactive subscale scores (*b* = 0.10, *p* < 0.001, *F*(3, 321) = 38.02, *p* < 0.001), somewhat smaller for the Investigative subscale scores (*b* = 0.07, *p* < 0.001, *F*(3, 322) = 20.93, *p* < 0.001) and non‐significant for Sensory subscale scores (*b* = 0.01, *p* = 0.092, *F*(3, 324) = 2.03, *p* = 0.120). Additionally, we found that the age effect on mean scores disappeared from the age of 13 months (*b* = 0.02, *p* = 0.087). These subscale patterns are in line with those for item difficulties and proportion of NA responses (Table 1), the latter of which also significantly decreased with age (*b* = −0.31, *p* < 0.001) but stabilized around the age of 14 months (*b* = −0.03, *p* = 0.066).

Lastly, the overall mean scores and the two curiosity items were positively correlated (direct item: *r*_*s*_ = 0.36, *p* < 0.001; comparative item: *r*_*s*_ = 0.21, *p* < 0.001), offering a first indication of construct validity. It is to be noted, however, that these single items are not validated measures themselves and therefore cannot reliably capture differences in curiosity (e.g., median value for the direct item was 7 out of 7). Other potential reasons for these lower correlations are that directly asking about curiosity is more prone to inflict response bias or that parents have a broader meaning of curiosity in mind when responding to these single‐item questions. Thus, these results may rather be considered as *initial* checks regarding the construct.

### 2.5 Interim Discussion

In this first study, we described the development of the ITCQ from creating an initial set of items capturing various behaviors with which infants and toddlers can explore and interact with their environment to the final set of 23 items comprising this internally consistent and structurally valid measure of trait curiosity. An initial pilot study provided evidence for the scale's content validity. The subsequent main analysis included 370 responses across an age range from 5 to 24 months and offered sufficient evidence to justify the computation of an overall mean score, along with three emergent subscales which could explain additional variance in the data. These subscales seemed to capture different types of curiosity manifesting in broader, sensory exploration, more focused, investigative exploration, and interacting with social others to gain information. While high internal consistency for the full scale further supported the computation of an overall curiosity score, good indicators for the subscales made these a valid option for exploratory analyses.

We then confirmed the scale's applicability across sexes and found that mean scores were only predicted by age but neither sex nor SES (but note that SES was rather homogenous in this sample). The age‐effect could be due to the scale items covering various exploratory behaviors which develop across the first 2 years of life and are thus bound to increase with time, making parental observations more robust and caregivers more confident in their reported agreement. The differential correlations between age and the subscales (Sensory < Investigative < Interactive), in line with patterns of NA responses and item difficulty, as well as the disappearance of this effect from 13 months of age onward lend support to this notion. Therefore, this effect should be controlled for in cross‐sectional studies including younger infants, for which the Sensory subscale would be most meaningful.

It is to be noted that a large number of responses concerned infants in the range of 10–12 months of age, which may have biased the item selection. This age group is somewhat in the middle of the full age‐range and part of the group in which age‐effects were observed, so that in fact, the selection would have been biased toward items that are applicable from earlier in development (making up around half of the scale as represented by the Sensory subscale). Nevertheless, longitudinal studies are needed to explore the developmental trajectory of item scores, for which we would expect to find rank stability (more curious children stay more curious) to support the scale's temporal consistency.

## 3 Study 2: Test‐Retest Reliability

Another important aspect of a scale's validation is test‐retest reliability which indicates the clarity of the items via the responses' temporal stability (Crocker and Algina 1986). If items are well constructed to capture observable behavior that reflects the child's general tendencies, the responses should be consistent with each other. Here, the retest timeframe was set to 7–14 days so that participants were unlikely to remember their previous responses and the child would not have experienced a leap in behavioral development.

### 3.1 Methods

#### 3.1.1 Participants

As mentioned previously, participants who provided consent and email address at the end of the survey were automatically contacted through Qualtrics 1 week after their initial response. From the participants included in the main analysis in Study 1, we collected *N* = 67 test‐retest responses completed within 7–14 days (*M* = 7.61, SD = 1.19) of the first measurement. Three additional responses were excluded due to longer timeframes (18, 46, and 144 days, respectively). Babies of the final 67 responders were typically developing and representative of the full sample (age in months at first timepoint: *M* = 12.7, SD = 5.1, range: 5.1–24.2; 58% female). Caregivers provided consent to proceed to the questionnaire items and completed this second measurement without any additional reward or compensation. Responses were matched via anonymous identification numbers, imbedded in the automatic emails.

#### 3.1.2 Materials

The test‐retest version of the full questionnaire included the original 36 items as well as the two curiosity questions. However, we conducted all analyses using only the final 23 items based on the results from Study 1.

#### 3.1.3 Analyses

Test‐retest reliability was investigated in two ways: how consistently participants responded to each item (using the function “testRetest” from the *psych* package (Revelle 2023)), as well as the Intraclass Correlation Coefficient (ICC) of mean scores (using the function “icc” from the *irr* package; Gamer, Lemon, and Singh 2019). The first analysis implemented the data's multi‐level structure to provide reliability indices for items and participants over time and indicated variance for each of these components and their interactions. Furthermore, we specified the ICC of mean scores as a two‐way mixed effect model with absolute agreement and single unit analysis as suggested by the literature (e.g., Koo and Li 2016). Historically, ICC scores have been considered as *poor* at values smaller than 0.5, as *moderate* between 0.5 and 0.75, as *good* between 0.75 and 0.9, and as *excellent* above 0.9.

### 3.2 Results

We found good internal consistency at both timepoints (T1: Coefficient ***α*** = 0.87, ***λ*** **‐6** = 0.94; T2: ***α*** = 0.88, ***λ*** **‐6** = 0.96), indicating that the items correlated with each other to a similar extent. Furthermore, item scores were correlated across measurements at *r* = 0.86 (*p* < 0.001). The mean within‐subject test‐retest reliability of response patterns over items and time was good (*rqq* = 0.79) as was the reliability of all ratings across items and times (*RkF* = 0.97) (Revelle 2023; Shrout and Lane 2012). Multilevel components of variance further showed that most of the variance in scores could be attributed to the items (44%), participants (13%), and the interaction between items and participants (23%). Little to no variance, however, was attributed to time effects (time: 0%; participant * time interaction: 0.1%; items*time interaction: 0%). This suggests that participants responded to items with sufficient temporal stability to support the scale's test‐retest reliability. A good ICC of mean scores seconded this finding (ICC(A, 1) = 0.82; *F*(66,47.9) = 11.3, *p* < 0.001; 95% CI = [0.72; 0.89]).

### 3.3 Interim Discussion

Both measures supported the scale's temporal stability indicating that the items were well constructed to allow for reliable responses. It is to be noted, however, that this sample was self‐selected and might represent highly motivated responders. Future research should aim to recruit a more representative sample and explore different timespans between measurements for a more in‐depth investigation.

## 4 Study 3: Criterion Validity

Another source of validity evidence is the measure's relationship to other variables (Downing 2003). This includes correlating them with scores of other existing measures with well‐known characteristics. Therefore, we decided to compare the new scale to facets of temperament as we would expect them to be related yet distinct. While the temperament scales mostly capture how the child typically reacts to certain situations, the ITCQ mostly captures infant‐initiated exploratory behaviors. As behavioral expressions may well be affected by how the child reacts to certain situations, we expected the temperamental facets to differentially correlate with the curiosity scores. Furthermore, it had previously been stated that an established relation between a curiosity measure and temperament would support the notion of it capturing curiosity as a trait (Piotrowski, Litman, and Valkenburg 2014).

Temperament is viewed as an early equivalent to adult personality traits, and its measures (Infant Behavior Questionnaire or IBQ; Early Child Behavior Questionnaire or ECBQ) have been shown to be reliable, valid, and informative both in personality related research but also for predicting behavioral outcomes (e.g., Putnam, Rothbart, and Gartstein 2008; Rothbart 1986, 2011), making them appropriate measures for exploring the ITCQ's criterion validity. In adults, positive accounts of curiosity similar to our conceptualization (e.g., Interest‐Type Epistemic Curiosity, Litman 2008; CEI Exploration Subscale, Kashdan et al. 2009) have been linked to higher Extraversion and Conscientiousness as well as lower Neuroticism (e.g., Hunter et al. 2016; Kashdan et al. 2018). Consequently, similar relations between the ITCQ and the equivalent temperament subscales would support the measure's validity and extend these findings into infancy.

### 4.1 Methods

#### 4.1.1 Participants

From the participants included in the main analysis in Study 1, *N* = 75 caregivers (children's age in months: *M* = 14.1, SD = 4.5, range: 6.5–24.2; 50.7% female) additionally completed the temperament survey, two thirds of which indicated having a degree in higher education. Most of these respondents were recruited directly from the Babylab's database, and thus, received £5 as reimbursement in the form of an online gift voucher of their choice (*n* = 55). The rest (*n* = 20) completed the temperament survey without additional rewards after their in‐person study visit for which they had received £5 travel reimbursement and a book for the child. Participants provided written consent prior to answering any questions.

#### 4.1.2 Materials

While the full temperament measure consists of around 200 items across multiple facets of temperament, the “very short form” versions (IBQ‐vsf and ECBQ‐vsf) each consists of 36 items evaluated on a 7‐point Frequency‐scale from 1 (“Never”) to 7 (“Always”) and an option of “NA—not applicable”, which have been validated to capture three broader dimensions: Surgency, Negative Affect, and Effortful Control (e.g., Putnam et al. 2010, 2014). Surgency items capture facets such as Approach, High Intensity Pleasure, Activity Level, and Perceptual Sensitivity, making this factor comparable to the personality dimension of *Extraversion*. Negative Affect items capture levels of Sadness, Distress to Limitations, and Fear, making this factor comparable to the personality dimension of *Neuroticism*. Lastly, Effortful Control items capture Duration of Orienting, and levels of Low Intensity Pleasure, Cuddliness, and Soothability, making this factor comparable to the personality dimension of *Conscientiousness*.

Participants first completed the full ITCQ, followed by either the very short form of the IBQ or ECBQ depending on the child's age: IBQ if the child was between 5 and 12 months old and the ECBQ for ages 13 months and over.

#### 4.1.3 Hypotheses

We expected Surgency to positively correlate with curiosity, as a more extraverted child may exhibit more exploratory behaviors across contexts. Second, we expected Negative Affect to negatively correlate with curiosity, as a more fearful and distressed child may exhibit fewer exploratory behaviors across contexts. Lastly, we did not have a clear prediction on how Effortful Control may correlate with curiosity but could hypothesize a positive relation with longer exploratory engagement in line with links found in adults (e.g., Hunter et al. 2016; Kashdan et al. 2018).

#### 4.1.4 Analyses

We computed mean scores for all scales (exploratorily also for the curiosity subscales) and conducted Spearman correlations between the temperament and curiosity scores. We treated scores from the IBQ and ECBQ equally, as items form into the same three dimensions and because of their assessed longitudinal stability (Putnam, Rothbart, and Gartstein 2008; Rothbart 1986).

### 4.2 Results

Correlations between the facets of temperament and curiosity are shown in Table 4. We found significant, positive correlations of moderate effect sizes between both surgency as well as effortful control and the mean curiosity score. Exploratory correlations revealed these to be strongest for the Sensory subscale. Additionally, we found a negative correlation between curiosity and negative affect. This relation seemed to be mainly driven by lower scores on the Interactive subscale so that young children reported to be more fearful and distressed were especially unlikely to interact with social others in the pursuit of information.

**TABLE 4:** Spearman correlation matrix between curiosity and temperament mean scores.

	Overall curiosity	Sensory	Investigative	Interactive
Surgency	0.39***	0.47***	0.31**	0.16
Negative affect	−0.27*	−0.24*	−0.14	−0.3**
Effortful control	0.25*	0.3**	0.17	0.08

***p < 0.001, **p < 0.01, *p < 0.05 with strongest correlations (≥ 0.3) in bold.

### 4.3 Interim Discussion

We investigated how the ITCQ related to other early traits measures, more specifically facets of temperament, to obtain evidence of its criterion validity. We found significant correlations of moderate effect size between all three temperament dimensions and overall curiosity in line with previous personality links found in adults (e.g., Hunter et al. 2016; Kashdan et al. 2018), whereas the subscales offered additional insights. The negative correlation between Curiosity and Negative Affect, that is, being more fearful and distressed, is furthermore in accordance with previous adult research that showed anxiety to be negatively associated with epistemic curiosity (Collins, Litman, and Spielberger 2004; Kashdan and Roberts 2004; Litman and Jimerson 2004; Litman and Spielberger 2003; Naylor 1981). Additionally, we observed the strongest negative correlation with the Interactive subscale which is consistent with the idea that Neuroticism may specifically inhibit social interactions and respective exploratory behaviors (Green and Campbell 2000). Together, these results provide evidence that curiosity and facets of temperament are related but still capture unique characteristics of the child's personality comparable to links found in adult personality research.

## 5 General Discussion

Recognizing the need to measure individual differences in trait curiosity in infants and toddlers, we developed the Infant and Toddler Curiosity Questionnaire (ITCQ) as the first caregiver report measure to assess trait curiosity in this targeted age group, with items capturing observable exploration behaviors specific to infants and toddlers between 5 and 24 months of age. Across three studies we reported evidence for the scale's reliability and validity, suggesting that the ITCQ could become a powerful tool for developmental research.

The first study focused on the initial questionnaire development leading to a final set of 23 items, selected based on internal consistency and exploratory factor analyses. Three methodologically emergent subscales captured additional co‐variance among the items and developmental exploration skills: sensory curiosity, investigative curiosity, and interactive curiosity as in gaining new information by interacting with social others. The well‐fitting correlational model using structural equation modeling and the strong correlations between subfactors offered sufficient support for the computation of an overall mean score. As the full scale but also each of the subfactors had good measures of internal consistency, we considered these subfactors as curiosity subscales. Furthermore, the scale seemed to work the same for male and female infants, but scores did increase with age until around 13 months. Together, this work offers multiple avenues to disentangle effects of trait curiosity but also of more specific types of curiosity manifestations. The second study then showed that the final scale had good test‐retest reliability after 7–14 days.

Lastly, study three indicated criterion validity as the ITCQ scores were significantly related to the well‐established trait measure of temperament (Putnam et al. 2014). Here, we found positive correlations between Curiosity and Surgency, which is considered a precursor of Extraversion, as well as Curiosity and Effortful Control, a precursor of Conscientiousness. In contrast, Curiosity negatively correlated with Negative Affect, a precursor of Neuroticism. Together, these findings are in line with theoretical considerations as well as previous personality research in adults (e.g., Collins, Litman, and Spielberger 2004; Hunter et al. 2016; Kashdan et al. 2018; Kashdan and Roberts 2004; Litman and Jimerson 2004; Litman and Spielberger 2003; Naylor 1981), providing crucial evidence for construct and criterion validity.

### 5.1 Limitations & Future Research

Questionnaire development is a strenuous process for which there is no gold‐standard as evidenced by numerous open discussions. Using the best practices as a guide, we created and assessed the ITCQ to be a reliable and valid measure (Downing 2003; Pett, Lackey, and Sullivan 2003). Yet, future studies are needed to collect multiple independent samples replicating these findings in more diverse socio‐economic populations and across different cultures. Even though the recruitment for the questionnaire study was largely conducted online via social media, and thus had the potential to reach a more representative population, the final sample ultimately was affected by a self‐selection bias of mainly highly educated, white responders. Consequently, the ITCQ's generalizability requires further investigation of potential socio‐cultural differences. For example, the encouragement of curiosity might be a privilege of those who have the time and resources, as well as the environmental safety to allow it. Studies in Tanzanian and Chinese parents, for example, have indicated that despite cultural norms emphasizing obedience over independence and curiosity, parental education and financial security were strong predictors of more positive and encouraging attitudes toward these constructs (Chuang and Su 2009; Jukes et al. 2021). Furthermore, it is possible that some items are not applicable across cultures in which children's interactions with social others to seek help and information are less pronounced (Little, Carver, and Legare 2016). However, this research avenue has already gained traction with planned investigations into cultural differences regarding the applicability of the ITCQ in non‐western cultures such as Japan, China, and India.

Another limitation concerns the necessity for longitudinal data to establish temporal stability of the trait measure, its developmental trajectories, and its convergent validity to other measures of early curiosity (Lee et al. 2023), problem solving (Hoicka et al. 2023) or observation‐based curiosity scores (Fortner‐Wood and Henderson 1997). Nevertheless, our reported studies here suggest that the ITCQ is a promising measure for application in psychological research to potentially explain variance in observed exploration behaviors (e.g., Mandler, Fivush, and Reznick 1987; Piccardi, Johnson, and Gliga 2020; Slone, Smith, and Yu 2019; Smith and Yu 2013) as well as developmental trajectories (e.g., Berg and Sternberg 1985; Bornstein, Hahn, and Suwalsky 2013; Muentener, Herrig, and Schulz 2018; Shah et al. 2018). In fact, preliminary reports of our measure have already gained international interest so that a German, Dutch, and Italian version of the ITCQ are currently undergoing validation, and a child version for 2–5‐year‐olds has also been developed (Altmann et al. 2023).

### 5.2 Conclusion

In this paper, we present the development of a newly constructed caregiver report questionnaire (ITCQ) and showcase that it effectively captures early exploration tendencies as a manifestation of individual differences in infants' and toddlers' trait curiosity. Importantly, the ITCQ fills an important gap in the scientific landscape of infancy research. Across three studies we demonstrated evidence for the measure's reliability and validity following rigorous practice to ensure that future applications of the ITCQ will offer new and powerful insights into early human development.

## Author Contributions

**Elena C. Altmann:** conceptualization, data curation, formal analysis, investigation, methodology, project administration, software, visualization, writing–original draft, writing–review & editing. **Marina Bazhydai:** conceptualization, funding acquisition, methodology, supervision, writing–review & editing. **Didar Karadağ:** conceptualization, methodology, writing–review & editing. **Gert Westermann:** conceptualization, funding acquisition, methodology, supervision, writing–review & editing.

## Conflicts of Interest

The authors declare no conflicts of interest.

## Supporting information

Supporting Information S1

## Data Availability

Data, materials, and analysis scripts presented in this manuscript are available from the OSF.
