# Supplementary material for: The Infant and Toddler Curiosity Questionnaire: A Validated Caregiver‐Report Measure of Curiosity in Children From 5 to 24 Months
Source: Infancy. 2025 Jan 23;30(1):e70001. doi: 10.1111/infa.70001 (PMC11758190; doi:10.1111/infa.70001)
Supplement: Supplementary file 1 — Supporting Information S1 [file INFA-30-0-s001.pdf]

Supplementary Materials

The r-script and raw data to reproduce the analyses, as well as a paper-pen-version for the questionnaire have been made available on the OSF.

1. Additional Items

1.1 Mirrored Items for attention

Ia6-M. When someone shows my child how something works, they are usually ***not*** very interested. *(Mirror of Final Item Ia6, Table 1; Can be additionally included in the middle of the item list, perhaps for online research with concerns over data quality, but must then be excluded from aggregate scores.)*

Ex1-M. My child usually waits to be given a toy to play with, rather than start playing by themselves. *(Mirror of excluded item Ex1, Table 1)*

1.2 Excluded Items (original numbering)

Excluded due to negatively correlating with the rest:

Item.5 When my child encounters an object, they are likely to put it in their mouth for further inspection (e.g., to see what it feels or tastes like).

Item.21 My child is usually happy to try new foods they haven't eaten before.

Excluded due to not loading onto the general factor:

Item.1 When I hold or move a toy or object in front of my child, they follow it with their eyes.

Item.4R When my child is introduced to something new, they are often not very interested.

Item.9 My child is constantly reaching for objects to explore.

Item.14 Once my child was able to crawl, they used this new skill to explore their environment on their own terms.

Item.29 R My child does not seem to care when we go somewhere new, they still prefer to engage with familiar objects they brought from home (e.g., their pacifier or favourite toy).

Item.36 My child is usually interested in new people.

Excluded due to not loading onto any subfactor:

Item.15/Ex1 My child starts playing on their own, rather than waiting to be given something to play with.

Item.18/Ex2 When my child plays with an assembly toy (e.g., building blocks, puzzle, a toy with detachable parts), they like to take it apart for further examination.

Item.25/Ex3 When playing hide and seek, my child enjoys searching for the object or person that disappeared.

1.3 Open Ended Questions

In reference to their response to the comparative item:

Please provide a short example that illustrates the option you just indicated. *(If more or less curious)*

Please provide a short example of how your child has recently (or indicate an approximate age) explored their environment in a way which was not reflected in the statements you rated. *(If equally curious)*

2. Additional considerations regarding the response scale

As the response scale we decided on a 7-point Likert scale from 1 (“strongly disagree”) to 7 (“strongly agree”). We considered using a frequency scale from *never* to *always* which is used in several infant and early childhood questionnaires (e.g., temperament scales IBQ and ECBQ). While both agreement (that is, Likert) and frequency scales can be used to generate aggregate scores, it was found in a systematic review that agreement scales lead to better fit and response quality and that frequency scales can be problematic in their interpretation (Brown, 2004). Thus, we decided on the format of agreement.

As the questionnaire was administered online using the secure software Qualtrics (Qualtrics, Provo, UT), we also had to consider in which way the response options would be presented. Next to the more conventional “radio-button” responses (one for each scale-point), a “slider” was discussed where caregivers could indicate their level of agreement anywhere between 0 and 100. While reviews suggest that such sliders can be more engaging with comparable data quality, they are in fact more time-intensive and can lead to frustration (Sikkel et al., 2014) and higher drop-out rates (Cook et al., 2001; Couper et al., 2006; Funke, 2016). Furthermore, they seem to add cognitive complexity which would not be recommended for wider representation of the caregiver population (Funke et al., 2011; Stanley & Jenkins, 2007). Based on these considerations, we decided to implement a conventional 7-point Likert scale with a button for each response option.

3. Number of exploratory factors

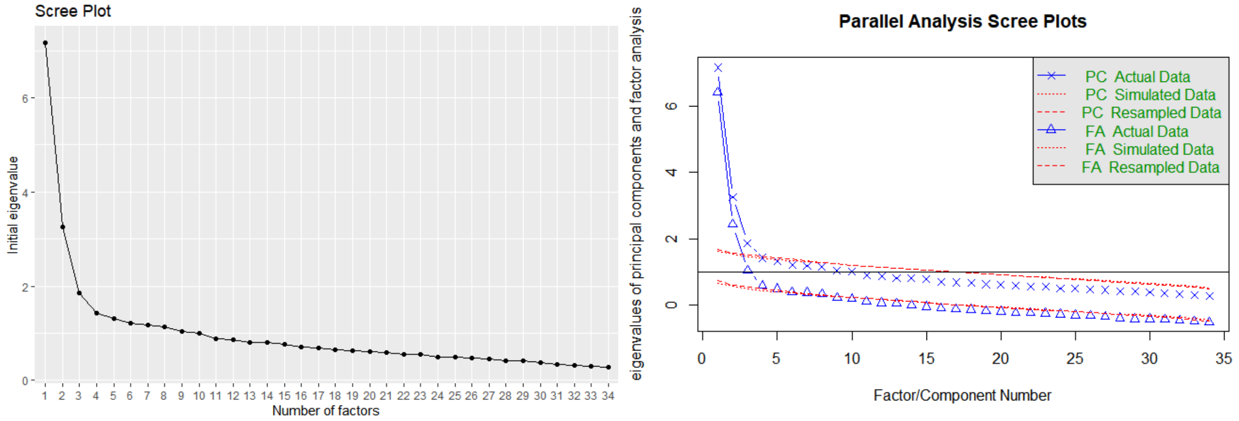

Figure S1. Scree plot & parallel analysis to determine the adequate number of factors suggesting 3 or 4 factors as indicated by their explanatory eigenvalues and the "elbow" in the graphs.

Supplementary Reference List

Brown, G. T. L. (2004). Measuring Attitude with Positively Packed Self-Report Ratings: Comparison of Agreement and Frequency Scales. *Psychological Reports*, *94*(3), 1015–1024. https://doi.org/10.2466/pr0.94.3.1015-1024

Cook, C., Heath, F., Thompson, R. L., & Thompson, B. (2001). Score Reliability in Webor Internet-Based Surveys: Unnumbered Graphic Rating Scales versus Likert-Type Scales. *Educational and Psychological Measurement*, *61*(4), 697–706. https://doi.org/10.1177/00131640121971356

Couper, M. P., Tourangeau, R., Conrad, F. G., & Singer, E. (2006). Evaluating the Effectiveness of Visual Analog Scales: A Web Experiment. *Social Science Computer Review*, *24*(2), 227–245. https://doi.org/10.1177/0894439305281503

Funke, F. (2016). A Web Experiment Showing Negative Effects of Slider Scales Compared to Visual Analogue Scales and Radio Button Scales. *Social Science Computer Review*, *34*(2), 244–254. https://doi.org/10.1177/0894439315575477

Funke, F., Reips, U.-D., & Thomas, R. K. (2011). Sliders for the Smart: Type of Rating Scale on the Web Interacts With Educational Level. *Social Science Computer Review*, *29*(2), 221–231. https://doi.org/10.1177/0894439310376896

Sikkel, D., Steenbergen, R., & Gras, S. (2014). Clicking vs. Dragging: Different Uses of the Mouse and Their Implications for Online Surveys. *Public Opinion Quarterly*, *78*(1), 177–190. https://doi.org/10.1093/poq/nft077

Stanley, N., & Jenkins, S. (2007). Watch What I Do! - Using graphical input controls in web surveys. *The challenges of a changing world, 81-92.*
